# Is reporting many cases of COVID-19 in Iran due to strength or weakness of Iran’s health system?

**Published:** 2020-04

**Authors:** Leila Mounesan, Sana Eybpoosh, Aliakbar Haghdoost, Ghobad Moradi, Ehsan Mostafavi

**Affiliations:** 1 Department of Epidemiology and Biostatistics, Research Centre for Emerging and Reemerging Infectious Diseases, Pasteur Institute of Iran, Tehran, Iran; 2 Modeling in Health Research Center, Institute for Futures Studies in Health, Kerman University of Medical Sciences, Kerman, Iran; 3 Social Determinants of Health Research Center, Research Institute for Health Development, Kurdistan University of Medical Sciences, Sanandaj, Iran

Newly identified discovered novel coronavirus disease (COVID-19) was identified at the end of 2019, when a cluster of pneumonia cases with an unknown cause was detected in Wuhan, China. Following the outbreak in china, new cases were also detected in other countries, and the epidemic continued to expand rapidly worldwide. Till date, COVID-19 has infected several thousands of individuals and has caused many fatal cases. Based on epidemiological evidence, the risk associated with COVID-19 epidemic in many countries is estimated to be moderate to high (1, 2). In March 11, 2020, the World Health Organization (WHO) announced COVID-19 as a pandemic. Currently, more than 600,000 confirmed cases of COVID-19 detected in 198 countries are reported worldwide (3).

**Fig. 1.**
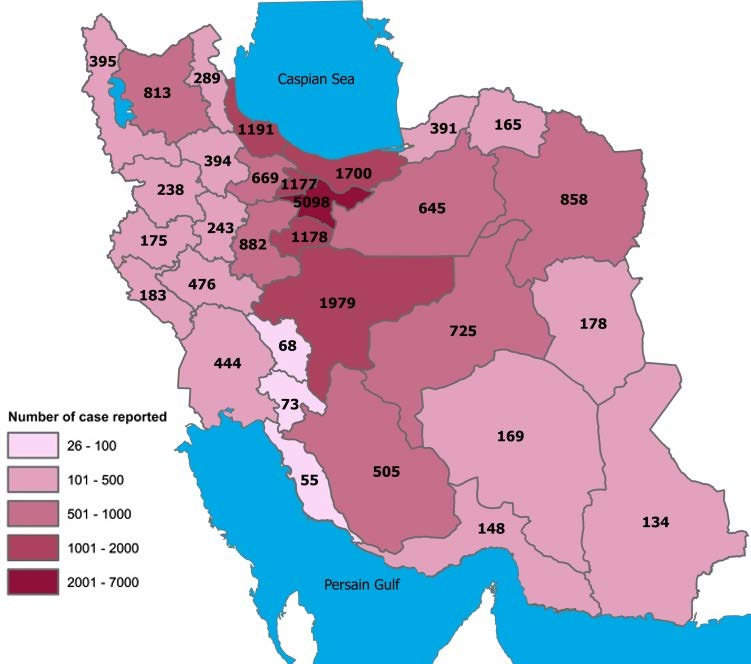
Geographical distribution of confirmed cases of COVID-19 in Iran, March 22, 2020 (4)

The first cases of COVID-19 in Iran, a country of eighty-three million people, were identified in February 19, 2020, when Real-Time Polymerase Chain Reaction (PCR) test of two cases who died in Qom turned positive for COVID-19. The surveillance system immediately scaled-up its case detection activities throughout the country. Samples were collected from all suspected cases referred to hospitals and tested for COVID-19 in the national reference laboratory. Within ten days, since reporting the first case of death in Iran, covid-19 had spread to 19 out of 31 provinces of the country, and Iran has become one of the global epicenters of COVID-19. By March 26, 2020, the number of laboratory-confirmed cases detected by Iran’s surveillance system reached 29406 cases with 2234 deaths and over 10457 recovered cases. Till now, the system has detected confirmed cases in all provinces of Iran (Fig. 1) (4, 5).

Prior to the detection of the disease in most countries of the world, Iran was faced with a major outbreak of COVID-19 and its reported cases were more than those reported by other countries in the Eastern Mediterranean region. Under similar conditions, a question is often asked: is reporting many cases due to strength or weakness of Iran’s health system? Is the outbreak in Iran really more dangerous, or perhaps Iranian health care system has been able to operate more efficiently?

The spread of the disease from China has been attributed to the level of its communication with other countries. Iran has communication with China in form of trade and traveling, however, the level of transfer between the two countries as well as the number of Iranians and Chinese living in the other country does not seem to be more than those observed in other countries of the region and the world. Hence, it can be concluded that the other countries are apparently infected through similar routes of communication at the same time or even sooner than Iran. Rapid reporting of cases of COVID-19 in Iran may be attributed to three factors: transparency in reporting cases, the importance of discovering high-risk hotspots, and the role of chance that can affect the rate of transmission and cause the faster discovery of the epidemic in a specific region. Concerning transparency, Iran has apparently tried to meet the standards and obligations.

To clarify why Iran’s health system was more sensitive toward COVID-19 outbreak, it is better to review Iran’s health infrastructure. Despite economic challenges imposed by sanctions, Iran has mobilized all its facilities and equipment for early detection, monitoring, and control of COVID-19. The major strengths of the health system in Iran include its public and private medical universities, primary, secondary, and tertiary care centers, and public and private laboratories, all of which are distributed as a network throughout the country. Ministry of Health with cooperation of Pasteur Institute of Iran is responsible for laboratory diagnosis of COVID-19. Currently, more than 50 laboratories in all provinces of Iran are providing diagnostic services in the country with the capacity to test more than 10’000 cases per day. The medical care services for cases with COVID-19 are provided by almost 750 hospitals, with more than 9000 beds (Hospital beds per 1000 population: 1.72, population/physician ratio: 900). The primary health care (PHC) network is the largest network of the health system in Iran, which provides access to primary healthcare in the most remote areas. Within the PHC network, there are 1,666 health posts, more than 5,000 rural and urban health centers, all of which are involved in the screening people, case follow-up, and contact tracing activities (6). In addition, public authorities have benefited from health volunteers by mobilizing more than 32,000 volunteer teams formed by the community and NGOs in order to provide a rapid and comprehensive response to COVID-19.

The 67 public medical universities in Iran has trained thousands of medical doctors, clinical specialists, nurses, and basic medical science scholars, most of whom have significantly contributed in the preparation of laboratories and primary, secondary, and tertiary medical centers to respond to COVID-19 outbreak. The epidemiology community of Iran, with hundreds of trained epidemiologists working in all provinces, is in charge of epidemic investigations in various zones and provision of technical consultation services and recommendations for regional and national health officials for a better control of the epidemic.

For years, Iran’s CDC have taken fundamental steps towards tackling important communicable diseases, such as polio, tuberculosis, malaria, HIV/AIDS, influenza, etc., and has learned valuable lessons from such efforts (7–9). Despite the country’s important achievements in tackling COVID-19, many areas still need urgent improvements. One of the major challenges is the bureaucracies and executive barriers toward cross-sectoral collaboration. One month after the detection of the epidemic in Iran, and despite intensive efforts made so far, there is still a need to significantly improve the participation of people in voluntary home isolation and social distancing. The response is now targeted toward these measures in order to provide a harmonized response and prevent the rapid growth of the epidemic. Iranian health system was expected to be susceptible to the epidemic and it was expected to detect the disease earlier, before the emergence of the first case of death, because a delay of a few weeks in early warning would result in catastrophic consequences. One of Iran’s challenges in dealing with the outbreak was managing data and presenting a sensitive case definition of the disease to detect more cases; the mentioned challenges caused problems in achieving accurate statistical data during the first two weeks of the epidemic. Although Iran reports the statistical data on a daily basis and tries to present the data and report transparently, the accuracy in case detection and reporting is not the same in all provinces, and there are still several reporting systems. Thus, sometimes there are inconsistencies in reported statistical data, and some indicators cannot be calculated precisely.

Although the epidemic condition is established in Iran, and the surveillance system is progressively detecting new cases each day, some of the countries in the region, with similar or better economic and development status, have detected a smaller number of cases of COVID-19 (Table 1). Eleven out of 22 countries in Eastern Meditation Region are classified as low, (n=6), or medium (n=5) development countries, and the status of some of the countries of the region is more complicated due to the war and refugee crisis. These countries mainly include Yemen, Syria, Iraq, and Afghanistan. Yemen is one of the few countries in the world that has not yet reported the disease. Due to socio-economic instabilities, most of these countries lack acceptable infrastructures required for giving a proper response to COVID-19 epidemic, including sensitive surveillance systems, adequate number of medical centers, and laboratory facilities, and COVID-19 outbreak can impose critical challenges on these countries.

**Table 1.** Some development criteria and the number of cases of COVID-19 in Eastern Mediterranean countries (11)

**Country**	**Population million (2015)**	Human Development Index* (2017)	**Life expectancy**		**Gross national income (GNI) per capita (2011 PPP $) (2017)**	**Cumulative no. of COVID-19 cases (Updated March 25, 2020)**		
	
			**Female**	**Male**		**Confirmed cases**	**Recovered cases**	**Deaths**
Afghanistan	35.5	LHD	63.2	63.9	0	75	2	1
Bahrain	1.5	VHHD	80.4	78.8	41,580	419	190	3
Djibouti	0.9	LHD	65.5	62.2	3,105	12	0	0
Egypt	84.7	MHD	74.4	68	10,355	442	93	21
Iran	81.2	HHD	79.4	76.5	19.130	27017	9625	2077
Iraq	38.3	MHD	78.8	74.8	17,789	346	89	29
Jordan	9.7	HHD	88.1	77.9	8,288	153	1	0
Kuwait	4.1	VHHD	87.2	80.7	70,524	195	43	0
Lebanon	6.1	HHD	80	75.8	13,378	333	8	4
Libya	6.4	HHD	75	71.2	11,100	1	0	0
Morocco	35.7	MHD	74.8	73.3	7,340	170	6	5
Oman	4.6	VHHD	79.5	75.5	36,290	99	17	0
Pakistan	197	MHD	67.5	66.4	5,311	991	18	7
Palestine	4.8	MHD	78	75.6	5.055	62	17	0
Qatar	2.6	VHHD	81.7	79.6	116,818	526	41	0
Saudi Arabia	32.9	VHHD	79.4	75.3	49,680	900	29	2
Somalia	14.74	LHD	57.3	53.7	7,480	1	0	0
Sudan	40.5	LHD	72.1	68.9	4,119	3	0	1
Syria	18.3	LHD	75	65.5	2,337	1	0	0
Tunisia	11.5	HHD	80.8	76.2	10,275	173	1	5
UAE	9.4	VHHD	77	77.1	67,805	333	52	2
Yemen	28.3	LHD	70.3	66	1,239	0	0	0

* Human Development Index is broken down into four tiers: VHHD: very high human development (0.8–1.0), HHD: high human development (0.7–0.79), MHD: medium human development (0.55–.70), and LHD: low human development (below 0.55).

Improved case detection is crucial for the control of the epidemic in every country. Undetected and poorly-controlled epidemics in a country would also affect other countries in a short period of time (10). Therefore, proper detection of COVID-19 is crucial for the control of the epidemic not only in every country, but also in other countries and the whole region.

Because of similarities between the countries of the region in terms of socio-cultural background, which highly affects populations’ behaviors and health and economic infrastructures, it is recommended that these countries further focus on strengthening their diagnostic capacities, community participation, and the utilization of the lessons learned in affected countries, especially those with similar socio-economic contexts.
